# Prediction of delayed recovery from pediatric community-acquired pneumonia

**DOI:** 10.1186/1824-7288-36-51

**Published:** 2010-07-29

**Authors:** Massimiliano Don, Francesca Valent, Mario Canciani, Matti Korppi

**Affiliations:** 1 Pediatric Care Unit, "Sant'Antonio" General Hospital, San Daniele del Friuli, Udine, Italy; 2 Hygiene Department, School of Medicine, DPMSC, University of Udine, Udine, Italy; 3 Pediatric Department, School of Medicine, DPMSC, University of Udine, Udine, Italy; 4 Pediatric Research Centre, Tampere University and University Hospital, Tampere, Finland

## Abstract

**Background:**

If children with community-acquired pneumonia (CAP) do not recover within 48 hours after starting antibiotic therapy, complications are possible and a checkup must be ensured.

Aim of the present study was to evaluate the improvement of pediatric CAP, within 48 hours after starting therapy, in relation to age, etiology, clinical/laboratory characteristics and selected antibiotics.

**Methods:**

Ninety-four children were treated for radiologically confirmed CAP, 64 by oral amoxicillin, 23 by intravenous ampicillin and 7 by other antibiotics. The etiology of CAP was studied by serology, data on more than 20 clinical characteristics were collected retrospectively, and antibiotics were selected on clinical grounds.

**Results:**

After starting of antibiotics, the mean duration of fever was higher in children ≥5 than <2 or 2-4 years of age (p = 0.003). Fever continued >48 hours in 4 (4.3%) children and 2 additional children had empyema. Clinical, radiological and laboratory characteristics and serological findings were not significantly associated with the duration of fever. Fever continued >24 hours in 1 (4.8%) child treated with ampicillin and in 2 (8%) inpatients compared with 19 (28.8%) children treated with amoxicillin (p = 0.007) and 23 (33%) outpatients (p = 0.0012), respectively.

**Conclusions:**

Respiratory rate and erythrocyte sedimentation rates were associated with rapid decrease of fever. Anyway, none of the reported characteristics was able to predict treatment failures or delayed fever decrease in children suffering from CAP.

## Background

*Streptococcus pneumoniae *is the most common bacterial agent in pediatric community-acquired pneumonia (CAP) at any age, while *Mycoplasma pneumoniae *is more common among patients over 5 years of age [1]. The majority, over 90%, of basically healthy, western children with CAP clinically improve with disappearance of fever and reduction of breathing work within 48 hours after the onset of antibiotics, with no significant differences between beta-lactames and macrolides as first-line antibiotics [2-4]. In recent years, pneumococcal macrolide resistance has become an emerging problem [1], and complications such as empyema and even necrotizing pneumonia have become more common than earlier, particularly complicating pneumococcal pneumonia [5-7]. Therefore, most international and national guidelines recommend penicillin, amoxicillin or other beta-lactames as first antibiotic choice for all children with CAP at <5 years of age, and also in older children if clinical signs and symptoms suggest pneumococcal etiology [1]. The "48 hours rule" has been included in most guidelines [8]: if symptoms and signs of pneumonia have not started to improve within 48 hours after the beginning of antibiotic therapy, the child must be re-evaluated and treatment must be changed if indicated.

We have recently published our results on the etiology of infection (determined by serological means), [9-11] the severity of illness (assessed by serum procalcitonin and need of hospital care) [12] and the value of clinical features [13] in differentiating between viral, pneumococcal and atypical bacterial infections in 101 children with CAP confirmed by radiology. In the present paper we report the outcome of these children, in relation to age, etiology, clinical characteristics and first-line antibiotics, with special focus on the improvement within 48 hours after starting therapy.

## Methods

### Study subjects

During a surveillance period of 15 months in 2001-2002, 101 consecutive, previously healthy children with signs and/or symptoms compatible with respiratory infection (fever ≥37°C, tachypnea, cough and/or findings of crackles, bronchial breathing or silenced sounds on auscultation) and radiological infiltrations consistent with pneumonia were eligible for the study at the Department of Paediatrics, University Hospital of Udine, Italy [9-13]. The exclusion criteria were the neonatal age, chronic infectious and non-infectious underlying diseases, wheezing on auscultation and hospital-acquired pneumonia [9]. The study was approved by the Ethics Committee of the University of Udine, School of Medicine, and an informed oral consent was obtained from the parents of all children. The time between the beginning of antibiotic therapy and the stable disappearance of fever, always accompanied by a substantial clinical improvement, was estimated from the fever curves of the patient records for inpatients, or by asking the outpatients' parents at the pediatrician's visit or by phone 48-72 hours after the diagnosis. In particular, the body temperature was taken in hospitalized patients by the nurses every six hours a day, while the outpatients' parents were specifically required to take and write the body temperature down, at home, at least twice a day, after a brief training in body temperature measuring received by the nurses at the time of diagnosis. In both in- and out-patients the body temperature was taken on the axillary level, by means of standard mercury thermometers, that were supplied to the outpatients' parents by the investigators. The body temperature data were available in 94/101 (93%) children, and they formed the subjects of the present study: their mean age was 4.7 years (range 0.3-14.7 years).

### Etiological studies

*Streptococcus pneumoniae *etiology of CAP was diagnosed by significant increases of IgG antibodies to pneumococcal pneumolysin or C-polysaccharide between paired sera, using enzyme immunoassay (EIA). Non-capsulated *Haemophilus influenzae *and *Moraxella catarrhalis *infections were diagnosed by significant increases of IgG antibodies to whole cell antigens using EIA; all cases were mixed infections with viruses or other bacteria. *Mycoplasma pneumoniae *etiology was diagnosed by significant increases of antibodies in either complement fixation (CF) or EIA serology. Correspondingly, *Chlamydia pneumoniae *etiology was diagnosed by EIA and/or by micro immunofluorescence (MIF) [9], and *Simkania negevensis *etiology by MIF [10]. Viral involvement by respiratory syncytial virus (RSV), human metapneumovirus (hMPV), influenza A and B virus, parainfluenza 1, 2 and 3 virus, adenovirus and cytomegalovirus was studied by EIA in paired sera [9,11].

### Laboratory and radiological studies

On admission, C-reactive protein (CRP) and procalcitonin (PCT) were measured in serum samples, white blood cells (WBC) and erythrocyte sedimentation rates (ESR) in blood samples, as described previously [12]. In 2005, three experienced radiologists interpreted the chest radiographs, and the infiltration was considered as alveolar in 63 cases and as interstitial in 38 cases [12]. Pleural fluid was present in 14 cases.

### Clinical definitions

Medical history and clinical characteristics were registered in the hospital records of the patients on admission by the medical doctors on duty. In 2006, one of the authors (MD) collected data on over 20 items from these records using a structured case record form, including age, gender, fever on admission, respiratory rate on admission, preceding and presenting clinical signs and symptoms, and findings on physical examination. "Tachypnoea" was defined by the World Health Organization criteria, that are respiratory rate >60 breaths/minute in children aged <2 months, >50 breaths/minute in children aged 2-12 months and >40 breaths/minute in children aged >12 months [14]. "Length of fever" was defined as the period in which a patient became spontaneously (without the use of antipyretic) and steadily non-feverish: the hours elapsed from the time of CAP diagnosis to the time a body temperature >37°C was last recorded. "Delayed recovery" was defined as the combination of those patients with fever for longer than 48 hours and/or those patients who developed a complication, such as pleural empyema or lung abscess [8].

### Pharmacological choices

According to the policy of the hospital, all children with pneumonia diagnosed by chest radiography were treated with antibiotics, and the preferred drugs were amoxicillin for oral therapy and ampicillin for intravenous therapy. Other antibiotics were chosen by the doctors on duty only by specific indications (cephalosporins in case of penicillin allergy, macrolides in case of high suspicion of atypical etiology). Oral amoxicillin was started for 55 children, oral amoxicillin-clavulanic acid for 4 children, intravenous ampicillin for 21 children, oral cephalosporin (cephalexin) for 2 children, intravenous cephalosporin (ceftriaxone) for 6 children, and oral macrolides for 4 children. Vancomycin was started in one and quinolones in no case. In both in- and out-patients, the first antibiotic dose was given at the time of diagnosis, making so easy the record of the starting time of such therapy.

Acetaminophen, iboprufen or ketoprofen were allowed and they were the most commonly prescribed antipyretic and analgesic drugs in case of high fever or pain, such as headache, chest pain, arthralgia, referred abdominal pain [8].

### Statistical analysis

In univariate statistical analyses, Fisher's exact test was used for discrete variables and analysis of variance for continuous variables. In multivariate analyses, adjusted for age, linear regression was used for continuous variables and stratified analyses with Mantel-Haenzel chi square tests for discrete variables.

## Results

Forty-eight patients (51%) were enrolled during the winter season, from December to March. Seventeen patients were <24 months old, 41 were 2 to 4 years old and 36 were ≥5 years old. Twenty-five children were treated in hospital, and their mean hospitalization time was 5.0 days (range 3-13 days). Bacterial infection was diagnosed in 45 patients; *S. pneumoniae *was found in 17, *M. pneumoniae *in 25, *C. pneumoniae *in 8 and *S. negevensis *in 5 cases (9 were dual bacterial infections). Viral infection was diagnosed in 38 children, including 15 RSV cases and 5 hMPV cases; 19 were viral infections alone and 19 were mixed infections with typical or atypical bacteria.

After starting of antibiotics, the mean ± SD duration of fever was 23.0 ± 19.2 hours, being 15.6 ± 13.4 in <2 years, 18.9 ± 14.9 in 2-4 years and 31.2 ± 23.1 hours in ≥5 years old children (p = 0.003). Fever decreased in 44 (47%) children within 12 hours, in 25 (27%) between 13-24 hours, in 21 (22%) between 25-48 hours, and in only 4 (4%) children after 48 hours. Fever continued for >24 hours in 10/58 (14.7%) children aged <5 years compared with 15/36 (41.7%) children aged ≥5 years (p = 0.0154).

No clinical sign or symptom showed any significant association with the duration of fever (Table 1). The doctors assessed 37 patients as "ill-looking" on admission, 19 (51.4%) of which were treated with parenteral antibiotics, that was used in 9 (15.8%) of the 57 "well-looking" patients. From the 37 "ill-looking" patients, only 2 (5.4%) had fever >24 hours vs. 23/57 (40.4%) of "well-looking" children (p = 0.001). In both groups, *S. pneumoniae *was found in 19% of the cases. Likewise, no finding in physical or radiological examination associated with the duration of fever, except for respiratory rate that was associated with rapid, not with delayed fever abatement (Table 1). Elevated ESR was associated with rapid decrease of fever. A similar tendency was seen also for elevated CRP, PCT and WBC, but the results did not reach statistical significance (Table 1).

**Table 1:** Duration of fever in 94 children with community-acquired pneumonia, in relation to fever and other clinical signs/symptoms on admission, findings in physical and radiological examination and four serum non-specific inflammatory markers.

	< 12 hours	12-24 hours	> 24 hours	p-value
	(N = 27)	(N = 42)	(N = 25)	
Fever (°C)	38.6 ± 1.3	38.7 ± 0.9	38.6 ± 0.9	0.86 §
Fever >37,5°C (N = 81)	21 (25.9)	39 (48.2)	21 (25.9)	0.19 #
Fever >39,5°C (N = 16)	7 (43.8)	7 (43.8)	2 (12.5)	0.23 #
Cough (N = 84)	26 (31.0)	36 (42.9)	22 (26.2)	0.39 #
Vomiting (N = 29)	8 (27.6)	12 (41.4)	9 (31.0)	0.81 #
Chest pain (N = 15)	3 (20.0)	8 (53.3)	4 (26.7)	0.72 #
Abdominal pain (N = 23)	6 (26.1)	11 (47.8)	6 (26.1)	0.95 #
Respiratory rate *	40.45 ± 11.7	37.5 ± 14.5	30.1 ± 12.5	**0.04 **§
Tachypnea † (N = 29)	10 (34.5)	15 (51.7)	4 (13.8)	0.31 #
Crackles ‡ (N = 47)	15 (31.9)	20 (42.6)	12 (25.5)	0.83 #
Dullness (N = 11)	2 (18.2)	7 (63.6)	2 (18.2)	0.37 #
Decreased breath sounds (N = 56)	13 (23.2)	27 (48.2)	17 (30.4)	0.16 #
Alveolar infiltration (N = 58)	18 (31.5)	26 (44.8)	14 (24.1)	0.19 #
Pleural fluid (N = 12)	5 (41.7)	4 (33.3)	3 (25.0)	0.74 #
CRP (mg/L)	146.2 ± 131.9	159.3 ± 118.0	102.1 ± 103.3	0.25 §
PCT (ng/mL)	14.1 ± 19.6	10.5 ± 12.9	5.9 ± 14.9	0.18 §
WBC (cells/μL)	20363 ± 9846	18343 ± 9148	15669 ± 6612	0.16 §
ESR (mm/h)	81.9 ± 29.3	68.5 ± 31.6	57.6 ± 33.9	**0.03 §**

Data are presented as means ± SD or number (percentage). * Data were missing for 18 children; † by age-specific WHO criteria; ‡ fine and medium-sized inspiratory crackles (or rales) included. CRP: C-reactive protein; PCT: procalcitonin; WBC: white blood cells; ESR: erythrocyte sedimentation rate.

§ Analysis of variance; # Fisher's exact test.

Ninety-four patients with adequate data available were classified into 4 microbiological groups. The pneumococcal group consisted of all 18 cases with *S. pneumoniae *etiology (including cases with viral and mycoplasmal co-infection). The atypical bacterial group consisted of 27 cases with *M. pneumoniae, C. pneumoniae *or *S. negevensis *etiology (including cases with viral co-infection; pneumococcal co-infections excluded). The viral group consisted of 19 viral cases with no pneumococcal or atypical bacterial co-infections, and the 30 cases with no serological findings formed the group of unknown etiology. The serological classification of CAP had no association with the duration of fever, and the result remained negative also in multivariate adjusted analysis (data not shown). Oral amoxicillin was started for 11 patients (61%) of the pneumoccal group, 16 patients (59%) of the atypical bacterial group and 19 patients (63%) of the viral group.

Intravenous ampicillin was more effective than oral amoxicillin or macrolide (Table 2); fever continued >24 hours in only 1 (4.8%), compared with 19 (28.8%) patients treated with amoxicillin (p = 0.007) or with those 2 (50.0%) treated with macrolide (p = 0.056). In multivariate analyses, adjusted for age category, fever lasted in mean 11.1 hours less in children treated with ampicillin, than in those treated with amoxicillin (p = 0.001). The treatment setting was significantly associated with the duration of fever (Table 2). In hospitalized children the duration of fever was shorter than in outpatients; the difference was significant also in the age-adjusted analyses.

**Table 2:** Duration of fever in relation to antibiotic treatment (amoxicillin vs. ampicillin) and treatment setting (inpatients vs. outpatients).

	**Amoxicillin**	**Ampicillin**	**p-value**	**Inpatients**	**Outpatients**	**p-value**
	(N = 59)*	**(N = 21)**		**(N = 25)**	**(N = 69)**	
				
Hours (h) of fever	25.2 ± 18.6	13.4 ± 11.3	**0.012**	15.3 ± 13.6	25.8 ± 20.3	**0.0056**
<12 h	11 (18.6)	13 (61.9)		14 (56.0)	13 (18.8)	
12-24 h	29 (49.5)	7 (33.3)	**<0.0001**†	9 (36.0)	33 (47.8)	**0.0012**§
>24 h	19 (32.2)	1 (4.8)		2 (8.0)	23 (33.3)	

Data are presented as means ± SD or number (percentage).

Amoxicillin was administered orally, ampicillin intravenously.

* Four children with oral amoxicillin-clavulanic acid included.

† Age-adjusted comparison between ampicillin and amoxicillin groups: p = 0.0006; age-looking-setting-adjusted comparison between ampicillin and amoxicillin groups: p = 0.0018.

§ Age-adjusted comparison between inpatient and outpatient groups: p = 0.0058.

Finally, there were 6 patients with delayed recovery; fever continued >48 hours in four >7-year-old children, and two 3-year-old girls were treated for empyema for >2 weeks in hospital (Table 3). On admission, four of these 6 children were "looking ill", and CRP was >80 mg/L in all cases. The other clinical and laboratory findings varied a lot on admission. When the 2 children with empyema and the only child with lung abscess were excluded from the analyses, the mean duration of antibiotic treatment was 7.4 ± 3.1 days in the 90 children with no severe complication. The mean duration of intravenous treatment with ampicillin was 3.3 ± 1.5 days.

**Table 3:** Clinical characteristics and outcome of the six children with community-acquired pneumonia and delayed recovery.

**Findings **	**Case 1**	**Case 2**	**Case 3**	**Case 4**	**Case 5**	**Case 6**
	
On admission						
	
Age (yrs), gender	10, girls	10, boy	7, boy	8, girl	3, girl	3, girl
Fever (°C)	37.5	39.5	39.2	39.5	37.5	40.3
Looking ill	no	Yes	yes	no	yes	yes
CRP (mg/L)	98	270	292	82	202	426
PCT (ng/mL)	0.20	0.54	33.8	0.40	18.4	33.0
WBC (cells/μL)	8.760	18200	31400	16250	27010	31400
ESR (mm/h)	26	132	70	6	94	95
Pneumonia	interstitial	alveolar	alveolar	interstitial	alveolar	alveolar
Primary antibiotics	macrolide	vancomycin	ampicillin	amoxicillin	ampicillin	ampicillin
	
In hospital						
	
Complications	pleural fluid	lung abscess	no	no	empyema	empyema
Fever >48 hours	yes	yes	yes	yes	no	no
Hospital stay	no	7 days	7 days	no	15 days	15 days
Serology	Mycoplasma	none	pneumococcus	mixed*	mixed†	none‡

* Parainfluenza viral, mycoplasmal and pneumococcal serology positive; † influenza A viral and mycoplasmal serology positive; ‡ Group A hemolytic streptococci grew in pleural fluid.

## Discussion

The main result of the present study was that children with CAP improved well with the recommended treatment by oral amoxicillin or intravenous ampicillin [1,8]. However, starting with intravenous ampicillin, in this study for three days in average, seemed to be a more effective practice than starting with oral amoxicillin. Different conclusions were reached by a recent randomized clinical multicentre trial in over 1700 African, Asian or South-American children hospitalized for severe CAP, showing an equivalent effect for oral amoxicillin and parenteral penicillin [15]. However, the cases with fever continuing for >48 hours were rather common (19%) in both groups. Instead, our observations are in line with the experiences from UK and Finland [4,6,16], where the treatment is often started with intravenous G-penicillin for an average period of one to three days, and then switched to oral amoxicillin. In these studies, over 90% of the children with CAP improved and were non-feverish within 48 hours, usually between 12 and 24 hours after the first dose of antibiotics [4,6]. The most recent equivalence study from UK, however, showed that oral amoxicillin and intravenous penicillin were equally effective in 246 children with CAP [17]. In the present study, all patients improved, and delayed improvement assessed by fever still present at 48 hours was low (<5%). In fact, as many as 75% of the patients were non-feverish as early as 24 hours after starting therapy.

Half of the 6 children with delayed decrease of fever had atypical bacterial etiology of infection, in line with earlier studies [18,19]. Overall, nearly 30% of the children had serological evidence of atypical bacterial etiology, and they improved well with no treatment with macrolides or quinolones. On the other hand, inappropriate selection of first line antibiotic may be the reason why, in the present study, the mean duration of fever was significantly higher in ≥5 years old children with high rates of atypical bacterial etiology and treated with macrolides, doxicycline or quinolones according to most guidelines [1,8]. However, delayed recovery was in no case due to viral infection, refractory to antibiotics, though viral involvement was demonstrated in >40% of the cases. Empyema needing surgical intervention was diagnosed in two children and pulmonary abscess treated conservatively in one child. Pleural fluid was present in one child with pneumococcal pneumonia, but *S. pneumoniae *was not serologically found in the two children with empyema. Thus, the rate of severe complications (3%) was low, in accordance with recent observations from Finland [6]. On the other hand, empyema and other complications after childhood pneumonia have been on increase in recent years in many countries [5,6]. The occurrence of severe complications in children with no underlying illness, although rare, stress the importance to make sure that substantial improvement of pneumonia starts within 48 hours by selected antibiotics [8].

We were not able to find, on admission, any useful clinical, radiological or laboratory factor in predicting delayed recovery or development of complications or, on the other hand, the recovery at the situation in which the treatment is selected appropriately. If non-beneficial outcomes are rare, as was the case in the present prospective study focusing on a wide severity spectrum of CAP, retrospective case-control studies have more statistical power. In a recent case-control study from Finland, duration of fever before admission, tachypnea on admission and pain on abdominal palpation were significant and independent risk factors in multivariate analyses for empyema in children with presumptive bacterial etiology of pneumonia [6]. In the present study, abdominal symptoms and tachypnea or dyspnea were common, but not predictive for delayed recovery, assessed by duration of fever. In the Finnish study, prolonged fever and persistence of high serum CRP during hospitalization were associated with the development of empyema [6]. In line, CRP was elevated in all our 6 CAP patients with delayed recovery.

Pneumococcal penicillin-resistance is rare, about 5%, in North Italy, but pneumococcal macrolide-resistance is as common as 25%; the figures are based on the monitoring of bacterial resistance to antibiotics in clinical samples (>600 samples from the Friuli Venezia Giulia region in 2004-2006). The Prospective Resistant Organism Tracking and Epidemiology for the Ketolide Telithromycin (PROTEKT) study [20] showed that about 40% of *S. pneumoniae *display multidrug-resistant phenotypes (resistance to three or more antibiotics), with highly variable prevalence rates observed in different countries; in particular, penicillin non-susceptibility and erythromycin resistance were 35.7% and 36.0%, respectively. Currently, no useful and rapid tests for pneumococcal infection are available for children in clinical practice. Determination of pneumococcal antigens in urine samples has been used in adults, but the tests have not been sufficiently sensitive and specific for clinical use in children with low rate of bacteremic pneumonia and high rate of pneumococcal carriage in nasopharynx [21]. In addition, mixed viral-pneumococcal infections and mixed mycoplasmal-pneumococcal infections are common, and co-infections with *S. pneumoniae *are difficult to diagnose [22].

In the present paper the diagnosis of pneumonia was radiologically confirmed in all cases, a large microbiological test panel was used, and patients represented the whole spectrum of pediatric CAP. The great diversity of etiologic agents in pediatric CAP makes the identification of risk factors difficult, which may result in one antibiotic (i.e. ampicillin) appearing more effective than another (i.e. amoxicillin). For example, children who are treated for pneumonia due to an atypical or viral infection by an ineffective empiric agent such as a beta-lactam, more likely will fail to improve within 48 hours, because of less effective therapy, not because of the agent itself. As described by Marchant et al. [23], this is a major confounder in clinical drug treatment and intervention trials.

## Conclusions

In conclusion, respiratory rate and erythrocyte sedimentation rates were associated with rapid decrease of fever, but none of the reported signs, symptoms, radiological or laboratory markers was able to predict delayed recovery or development of complications in children with CAP. According to current recommendations, *S. pneumoniae *should be always covered in the treatment of CAP in children, even in the case of proven viral or atypical bacterial pneumonia, as was done in >95% of the cases of the present study. An intravenous induction of antibiotics seemed to increase the efficacy of therapy, though the patients treated intravenously were selected on clinical grounds, and thus they were more severely ill [8].

## Competing interests

The authors of the present paper declare that the manuscript has been done without any financial support and that they have no competing interests.

## Authors' contributions

All the four authors have taken full responsibility for the paper and have read and approved its submission. MD is the first author, recruited all the patients and did all the samplings. FV performed the statistical analysis. MC and MK are the scientific persons in charge of the present study. MK gave a significant assistance in the drafting of the paper.

## Authors' information

MD was born in Udine, Italy, in 1973 and he received his Medical Doctor Degree at the University of Udine in March 1998 with top grades. In the same year he started his pediatric residency at the Department of Pediatrics of the University Hospital of Udine (head Prof. Alfred Tenore) and, after a regular five-year residency program, he received his degree as specialist with full marks *cum laude *(November 2003). His Specialization Thesis, centred on the etiological, metabolic and clinical topics of pediatric community-acquired pneumonia, gave rise to the publication of many papers in English language in international Journals. MD recently prepared a Research Doctoral Thesis on the published data of such "Pneumonia Project" and publicly discussed it in Tampere - Finland, August 2009 (supervisor Prof. Matti Korppi, opponent Prof. Jussi Mertsola), thus achieving the Academic title of Doctor in Medical Science (MD, PhD). The data showed in the present paper directly derive from that "Pneumonia Project". MD currently works as attending physician at the Pediatric Care Unit of the "S. Antonio" General Hospital, San Daniele del Friuli - Udine - Italy (head Dr. Bruno Sacher).
